# Long‐term changes in psoas muscle mass after lobectomy and segmentectomy for early‐stage lung cancer

**DOI:** 10.1002/jcsm.13328

**Published:** 2023-09-23

**Authors:** Tetsuya Isaka, Hiroyuki Ito, Tomoyuki Yokose, Haruhiro Saito, Hiroto Narimatsu, Hiroyuki Adachi, Jun Miura, Kotaro Murakami, Noritake Kikunishi, Naoko Shigeta, Yasushi Rino

**Affiliations:** ^1^ Department of Thoracic Surgery Kanagawa Cancer Center Yokohama Japan; ^2^ Department of Surgery Yokohama City University Yokohama Japan; ^3^ Department of Pathology Kanagawa Cancer Center Yokohama Japan; ^4^ Department of Thoracic Oncology Kanagawa Cancer Center Yokohama Japan; ^5^ Department of Genetic Medicine Kanagawa Cancer Center Yokohama Japan; ^6^ Cancer Prevention and Cancer Control Division Kanagawa Cancer Center Research Institute Yokohama Japan; ^7^ Graduate School of Health Innovation Kanagawa University of Human Services Kawasaki Japan

**Keywords:** Less invasive, Lobectomy, Psoas muscle mass, Psoas muscle area, Sarcopenia, Segmentectomy

## Abstract

**Background:**

Segmentectomy is considered a less invasive procedure than lobectomy for patients with non‐small cell lung cancer (NSCLC); however, little is known about the physiological mechanism underlying the lower invasiveness of segmentectomy. This study is aimed to compare the differences in the long‐term changes in the psoas muscle mass after segmentectomy and lobectomy in patients with NSCLC.

**Methods:**

Overall 315 recurrence‐free patients who underwent segmentectomy (*n* = 93) or lobectomy (*n* = 222) for clinical stage 0‐I NSCLC between January 2016 and December 2018 and underwent computed tomography during the entire period of 6 months ≤ postoperative year (POY) 0.5 < 12 months, 12 months ≤ POY 1 < 24 months, 24 months ≤ POY 2 < 36 months, and 36 months ≤ POY 3 < 48 months were included. Bilateral psoas muscle area (PMA) at the L3 level was measured using each cross‐sectional computed tomography scan. Differences between the segmentectomy and lobectomy groups in the mean change of postoperative PMA from the preoperative period were analysed using Student's *t*‐test and mixed analysis of variance. Multivariable analysis was performed to identify the risk factors for PMA loss on POY 3 using logistic regression analysis.

**Results:**

The lobectomy group had a significantly larger PMA change than the segmentectomy group during each postoperative period (*P* < 0.001). Mixed analysis of variance revealed that the mean PMA change was significantly smaller in the segmentectomy group than in the lobectomy group during the observation period (*P* < 0.001). The mean change in the PMA was significantly larger from POY1 (−2.5%) to POY2 (−3.9%) and POY3 (−4.7%) in the lobectomy group (*P* = 0.003 and *P* < 0.001). However, PMA remained unchanged during the postoperative observation period in the segmentectomy group. In the multivariable analysis, the risk factors for PMA change ≤−3.3% (cut‐off: mean change of PMA) at POY3 included lobectomy [odds ratio (OR), 3.32; 95% confidence interval (CI), 1.90–5.82; *P* < 0.001], male sex (OR, 1.92; 95% CI, 1.02–3.62; *P* = 0.044) and open thoracotomy (OR, 1.84; 95% CI, 1.11–3.05; *P* = 0.017). After propensity score matching, the mean change in PMA was smaller in the segmentectomy group (*n* = 75) than in the lobectomy group (*n* = 75) during the postoperative observation period (*P* < 0.001).

**Conclusions:**

Psoas muscle mass was better maintained during the postoperative period by segmentectomy than by lobectomy. Psoas muscle mass reduction progressed over a long postoperative period after lobectomy. Segmentectomy via complete video‐assisted thoracic surgery is associated with a lower likelihood of sarcopenia progression.

## Introduction

Lobectomy is the standard procedure for resectable primary lung cancer.^1^ However, segmentectomy is now reported to be an acceptable procedure for early‐stage primary lung cancers.^2^, ^3^, ^4^, ^5^ In a recent JCOG 0802/WJOG4607L randomized trial comparing the overall survival (OS) after segmentectomy and lobectomy for non‐small cell lung cancer (NSCLC) ≤ 2 cm and consolidation/tumour ratio > 0.5, segmentectomy was superior to lobectomy after resection. The lower incidence of non‐lung cancer deaths in the segmentectomy group contributed to the better OS of segmentectomy than lobectomy.^4^ The results showed that segmentectomy was a less invasive procedure for patients with NSCLC than lobectomy; however, the physiological mechanism of the lower invasiveness of segmentectomy for patients remains unclear.

Several meta‐analyses have reported that segmentectomy preserves respiratory function better than lobectomy.^6^, ^7^ The mean decrease in forced expiratory volume in 1 s (FEV1) within 2 and 12 months of surgery was small in segmentectomy (−18% and −5%, respectively) compared with lobectomy (−25% and −11%, respectively).^8^ The JCOG 0802 trial showed that segmentectomy was 2.7% better than lobectomy in terms of FEV1 at 6 months and 3.5% better at 1 year.^4^ However, several studies have described that the advantage of preserving respiratory function in segmentectomy diminishes in the long‐term period after surgery.^9^, ^10^ Further investigation of the physiological markers that explain the invasiveness of segmentectomy in the long‐term period after surgery is necessary.

Sarcopenia is muscle failure resulting from muscle deterioration that occurs across a lifetime and shortens people's life expectancy by impairing physiological functions.^11^ The psoas muscle area (PMA) at the L3 level by computed tomography (CT), which represents psoas muscle mass, is known to be a surrogate for sarcopenia and skeletal muscle mass^12^ because of a strong linear relationship between whole‐body muscle mass and the cross‐sectional area of the psoas muscle mass on CT.^13^ Lower psoas muscle mass or skeletal muscle mass at the L3 level in the preoperative period was associated with postoperative complications of lung^14^ and other cancers.^15^, ^16^ Furthermore, they have been associated with postoperative prognosis in lung,^14^, ^17^, ^18^, ^19^ rectal,^20^ head and neck^21^ and breast cancers.^22^

Deterioration of the skeletal muscle leads to sarcopenia, which results in physiological dysfunction, organ damage, chronic inflammation, cachexia, age‐related loss of muscle mass and worsening patient survival.^11^, ^23^ The present study focused on long‐term changes in the psoas muscle mass after segmentectomy and lobectomy to determine whether segmentectomy could have a preventive effect on the development of sarcopenia. Moreover, the risk factors for the loss of the psoas muscle mass in the long‐term postoperative period were analysed.

## Methods

### Patients and surgical procedures

A total of 446 consecutive recurrence‐free patients underwent segmentectomy and lobectomy at our hospital between January 2016 and December 2018 for clinical stage 0‐I NSCLC without prior lung resection. Among these, 315 patients (222 patients with lobectomy and 93 patients with segmentectomy) who underwent CT during the entire postoperative period of 6 months ≤ postoperative year (POY) 0.5 < 12 months, 12 months ≤ POY1 < 24 months, 24 months ≤ POY2 < 36 months, 36 months ≤ POY3 < 48 months, and preoperative period (POY0) were included in this study.

Patients who underwent two segmentectomies or a subsegmentectomy in addition to one segmentectomy with a smaller extent of lung resection than lobectomy were included in the segmentectomy group. Patients who underwent bilobectomy or lobectomy in addition to one segmentectomy were excluded from the study. Segmentectomy was performed for ground‐glass opacity‐dominant lung tumours ≤ 2 cm in diameter that could not be removed with sufficient margins using wedge resection. Lobectomy was performed for solid‐dominant lung tumours or ground‐glass opacity‐dominant tumours that could not be removed with sufficient margins using wedge resection or segmentectomy. Segmentectomy was performed in patients who could not tolerate lobectomy due to advanced age, low pulmonary function or many other complications.

An open thoracotomy was performed through a posterolateral or anterior axillary incision. Complete video‐assisted thoracoscopic surgery (cVATS) was performed with a 3.0–3.5 cm utility incision in the fifth or sixth intercostal space in the middle axillary line and three additional ports approximately 1 cm in length. The selection of open thoracotomy or cVATS was made at the discretion of the surgeon.

### Patient follow‐up

CT of the thorax and upper abdomen was performed every 6**–**12 months for 5 years postoperatively. In addition, blood tumour markers and chest radiography were performed every 3**–**6 months for 3 years postoperatively and every 6**–**12 months for 3**–**5 years at an outpatient facility. Positron emission tomography‐CT and head magnetic resonance imaging were performed if lung cancer recurrence was suspected. Treatment strategies after recurrence were decided at a joint conference consisting of thoracic surgeons, respiratory physicians, pathologists and radiologists.

### Psoas muscle area and latissimus dorsi muscle area measurements and calculation of the reductions

Bilateral PMA at the L3 level were measured in the cross‐section of CT using a region‐of‐interest measurement tool. Bilateral latissimus dorsi muscle areas (LMA) at the Th4 level were also measured. Each bilateral muscle area was traced manually and automatically using the SYNAPSE VINCENT software (Fujifilm Medical Co., Ltd., Tokyo, Japan). Each muscle was identified and quantified using a mediastinal window level setting (level 45 Hounsfield unit [HU]; width 300 HU) in a 10 mm section thickness. PMA and LMA were calculated by averaging the area of each muscle measured on CT by T. I. and H. A. under conditions in which the patients' information was blinded. All preoperative CT scans were performed within 2 months of surgery. If multiple CT scans were performed during POY0.5, POY1, POY2 and POY3, the area of each muscle was measured using the CT performed during the period closest to the date of surgery. The percentage changes in PMA and LMA were calculated as follows:

$$\text{The}\;\text{percentage}\;\text{of}\;\text{change}\;\text{of}\;\mathrm{PMA}\;\text{or}\;\mathrm{LMA}\;\left(\%\right)=\frac{\left(\text{postoperative}\;\mathrm{PMA}\;\text{or}\;\mathrm{LMA}\right)-\left(\text{preoperative}\;\mathrm{PMA}\;\text{or}\;\mathrm{LMA}\right)}{\text{preoperative}\;\mathrm{PMA}\;\text{or}\;\mathrm{LMA}}\times 100$$

### Statistical analyses

All statistical analyses were performed using EZR on R Commander, version 1.30 (Saitama Medical Center, Jichi Medical University, Saitama, Japan), a graphical user interface for R (The R Foundation for Statistical Computing, Vienna, Austria). Mann–Whitney *U* test or Student's *t*‐test was performed to compare continuous variables between groups, while Fisher's exact test was used to compare categorical variables. One‐way repeated measures analysis of variance (ANOVA) was performed to analyse the temporal PMA change at POY0.5, POY1, POY2 and POY3 from the postoperative period. Bonferroni adjustment was used for between‐group comparisons. Mixed ANOVA with two‐way repeated measures ANOVA using EZR on R Commander was performed to compare the mean changes in the PMA and LMA from the preoperative period between the lobectomy and segmentectomy groups (Supporting Information S1). The risk of PMA and LMA reduction at POY0.5 and POY3 were analysed by multivariable analysis using logistic regression analysis with the following variables: age (≧65), sex, body mass index (≦18.5 kg/m^2^), smoking history, FEV1/forced vital capacity ratio (FEV1%) < 70%, percentage of predicted vital capacity <80%, other cancer histories, co‐morbidities (chronic obstructive pulmonary disease, coronary disease, Charlson co‐morbidity index ≧1), postoperative complications, lobectomy, open thoracotomy and adjuvant chemotherapy. Postoperative complications were defined as grade II or higher based on Clavien–Dindo classification. The OS between the two groups was analysed using Kaplan–Meier method and compared using log‐rank tests. The mean changes of PMA and LMA at POY0.5 and POY3 were used as the cut‐off values. To reduce selection bias between patients who underwent lobectomy and segmentectomy, propensity score matching was used (1:1 matching method, calliper = 0.01) (Supporting Information S2). Statistical significance was set at *P* < 0.05.

## Results

The median follow‐up period was 57.3 months. Details of lobectomy and segmentectomy are shown in *Table* S1. The segmentectomy group had higher Charlson co‐morbidity index, frequencies of coronary disease, frequency of pathological stage I ≥ and smaller tumour size than the lobectomy group (*Table* 1). The lobectomy group had a significantly larger PMA change than the segmentectomy group during each postoperative period (*P* < 0.001; *Table* 1). However, there was no difference in the LMA change between the lobectomy and segmentectomy groups in each postoperative period (*Table* 1). The number of patients with PMA change ≤−2.0% at POY0.5 and PMA change ≤−3.3% at POY3 was significantly smaller in the segmentectomy group (both *P* < 0.001). However, there were no significant differences between the two groups in the number of patients with LMA change ≤−6.3% at POY0.5 and LMA change ≤−6.4% at POY3 (*P* = 0.621 and *P* = 1.000, respectively) (*Table* 1).

**Table 1:** Comparison of patient characteristics between lobectomy and segmentectomy groups

Total (*n* = 315)	Lobectomy (*n* = 222)	Segmentectomy (*n* = 93)	*P* values^a^
Age, mean (SD), year	67.8 (9.4)	67.6 (10.8)	0.507^b^
Male, No. (%)	106 (47.7)	43 (46.2)	0.902
Height, mean (SD), cm	159.4 (9.0)	159.4 (8.2)	0.985^c^
Weight, mean (SD), kg	57.4 (11.4)	58.5 (10.4)	0.423^c^
Body mass index, mean (SD), kg/m^2^	22.5 (3.4)	22.9 (3.0)	0.284^c^
Smoking history, No. (%)	115 (51.8)	58 (62.4)	0.106
FEV1%, mean (SD), %	73.6 (8.9)	72.6 (8.8)	0.565^b^
%VC, mean (SD), %	109.9 (14.7)	109.1 (15.5)	0.678^c^
CT tumour size >2.0 cm, No. (%)	136 (61.3)	28 (30.1)	<0.001
Other cancer history, No. (%)	50 (22.5)	29 (31.2)	0.118
Co‐morbidity, No. (%)			
Chronic obstructive pulmonary disease	15 (6.8)	7 (7.5)	0.811
Interstitial pneumonia	3 (1.4)	1 (1.1)	1.000
Coronary disease	7 (3.2)	8 (8.6)	0.046
Hypertension	73 (32.9)	37 (39.8)	0.247
Diabetes mellitus	22 (9.9)	16 (17.2)	0.087
Charlson co‐morbidity index, mean (SD)	0.8 (1.0)	1.2 (1.3)	0.019^b^
cVATS, No. (%)	85 (38.3)	35 (37.6)	1.000
Postoperative complications ≥G2, No. (%)	53 (23.9)	21 (22.6)	0.885
Adenocarcinoma, No. (%)	202 (91.0)	83 (89.2)	0.675
Pathological stage I ≥, No. (%)	204 (91.9)	92 (98.9)	0.017
Adjuvant chemotherapy, No. (%)	34 (15.3)	0 (0)	<0.001
Mean PMA change at POY0.5, mean (SD), %	−3.2 (5.9)	0.8 (6.2)	<0.001^c^
Mean PMA change at POY1, mean (SD), %	−2.5 (6.1)	1.9 (5.9)	<0.001^c^
Mean PMA change at POY2, mean (SD), %	−3.9 (7.3)	0.1 (7.3)	<0.001^c^
Mean PMA change at POY3, mean (SD), %	−4.7 (8.6)	0.0 (8.8)	<0.001^c^
Mean LMA change at POY0.5, mean (SD), %	−6.7 (10.6)	−5.4 (8.7)	0.304^c^
Mean LMA change at POY1, mean (SD), %	−5.2 (9.8)	−4.8 (9.8)	0.755^c^
Mean LMA change at POY2, mean (SD), %	−6.0 (11.7)	−5.3 (9.1)	0.600^c^
Mean LMA change at POY3, mean (SD), %	−6.8 (11.8)	−5.5 (9.2)	0.369^c^
PMA change ≦−2.0 at POY0.5, No. (%)	124 (55.9)	25 (26.9)	<0.001
LMA change ≦−6.3 at POY0.5, No. (%)	118 (53.2)	46 (49.5)	0.621
PMA change ≦−3.3 at POY3, No. (%)	121 (54.5)	27 (29.0)	<0.001
LMA change ≦−6.4 at POY3, No. (%)	115 (51.8)	48 (51.6)	1.000

CT, computed tomography; cVATS, complete video‐assisted thoracoscopic surgery; FEV1%, forced expiratory volume in 1 s/forced vital capacity ratio; LMA, latissimus dorsi muscle area; PMA, psoas muscle area; POY, post‐operative year; SD, standard deviaion; VC, vital capacity.

^a^ Fisher's exact test.

^b^ Mann–Whitney *U* test.

^c^ Student *t*‐test.

**Figure 1:**
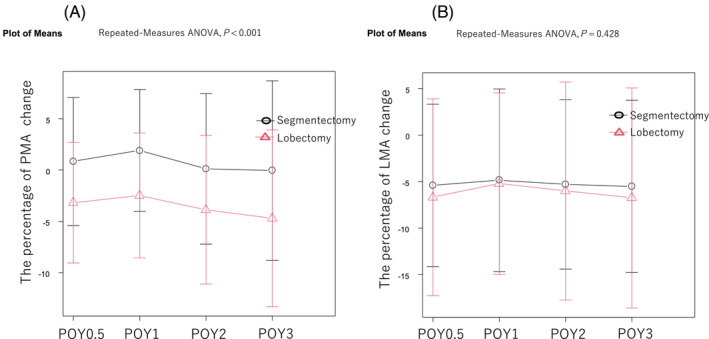
Comparison of PMA (A) and LMA (B) changes after lobectomy and segmentectomy. The mean PMA change during the postoperative observation period was significantly smaller in the segmentectomy group than in the lobectomy group (*Figure* 1A). Postoperative mean LMA change during the postoperative period in patients who underwent segmentectomy was not significantly different from that in those who underwent lobectomy (*Figure* 1B). ANOVA, analysis of variance; LMA, latissimus dorsi muscle area; PMA, psoas muscle area; POY, postoperative year.

Mixed ANOVA revealed that the postoperative PMA change was significantly smaller in the segmentectomy group than in the lobectomy group during the postoperative observation period (*P* < 0.001, *Figure* 1A). However, the postoperative LMA changes during the observation period in patients who underwent segmentectomy were not significantly different from those in patients who underwent lobectomy (*P* = 0.428, *Figure* 1B).

**Figure 2:**
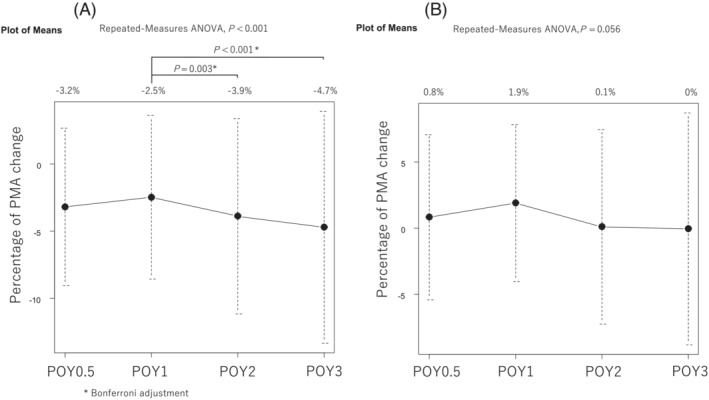
Postoperative change of PMA after lobectomy (A) and segmentectomy (B). The mean PMA change was significantly larger from POY1 to POY2 and POY3 in the lobectomy group (*Figure* 2A). The overall mean change in postoperative PMA was not significantly different during the postoperative observation period in the segmentectomy group (*Figure* 2B). ANOVA, analysis of variance; PMA, psoas muscle area; POY, postoperative year.

One‐way repeated measures ANOVA showed that the mean change of the PMA was significantly larger from POY1 (−2.5%) to POY2 (−3.9%) and POY3 (−4.7%) in the lobectomy group (*P* = 0.003 and *P* < 0.001, *Figure* 2A). The overall mean postoperative PMA change was not significantly different during the postoperative observation period in the segmentectomy group (*P* = 0.056; *Figure* 2B).

In the multivariable analysis, lobectomy was an independent risk factor for PMA change ≦−2.0% at POY 0.5 [odds ratio (OR), 3.47; 95% confidence interval (CI), 1.98–6.08; *P* < 0.001] (*Table* 2). Lobectomy (OR, 3.32; 95% CI, 1.90–5.82; *P* < 0.001), male sex (OR, 1.92; 95% CI, 1.02–3.62; *P* = 0.044) and open thoracotomy (OR, 1.84; 95% CI, 1.11–3.05; *P* = 0.017) were independent risk factors for PMA change ≦−3.3% at POY3 (*Table* 2). Open thoracotomy and postoperative complications were independent risk factors for LMA change ≤−6.3% at POY0.5 and LMA change ≤−6.4% at POY3; however, lobectomy was not an independent risk factor for LMA change ≤−6.3% at POY0.5 (OR, 1.12; 95% CI, 0.65–1.95; *P* = 0.685) and LMA change ≤−6.4% at POY3 (OR, 1.04; 95% CI, 0.61–1.78; *P* = 0.879) (*Table* 3).

**Table 2:** Risk factors for psoas muscle area reduction

Logistic regression model	PMA change ≦−2.0%^a^ at POY0.5			PMA change ≦−3.3%^b^ at POY3		
Variable	OR	95% CI	*P* values	OR	95% CI	*P* values
Age ≥65	0.83	0.48–1.44	0.505	1.01	0.58–1.76	0.978
Male	1.50	0.80–2.80	0.210	1.92	1.02–3.62	0.044
Body mass index ≤18.5 kg/m^2^	0.68	0.28–1.64	0.391	1.89	0.79–4.54	0.155
Smoking history	0.70	0.38–1.31	0.268	0.76	0.41–1.43	0.395
FEV 1% < 70%	0.87	0.49–1.55	0.640	0.85	0.48–1.51	0.568
%VC < 80%	1.15	0.18–7.40	0.883	1.20	0.18–8.04	0.848
Other cancer history	1.10	0.55–2.19	0.785	1.19	0.60–2.37	0.618
Chronic obstructive pulmonary disease	0.84	0.30–2.34	0.741	1.12	0.41–3.06	0.829
Coronary disease	0.61	0.81–2.03	0.417	1.32	0.42–4.19	0.634
Charlson co‐morbidity index ≧1	1.41	0.74–2.68	0.300	0.89	0.47–1.69	0.723
Lobectomy (vs. segmentectomy)	3.47	1.98–6.08	<0.001	3.32	1.90–5.82	<0.001
Open thoracotomy (vs. cVATS)	1.23	0.75–2.02	0.419	1.84	1.11–3.05	0.017
Postoperative complications ≥G2	0.94	0.54–1.63	0.818	0.87	0.50–1.51	0.618
Adjuvant chemotherapy	0.94	0.42–2.07	0.873	0.51	0.23–1.13	0.096

CI, confidence interval; cVATS, complete video‐assisted thoracoscopic surgery; FEV1%, forced expiratory volume in 1 s/forced vital capacity ratio; OR, odds ratio; PMA, psoas muscle area; POY, post‐operative year; VC, vital capacity.

^a^ Mean PMA change at POY0.5.

^b^ Mean PMA change at POY3.

**Table 3:** Risk factors for latissimus dorsi muscle area reduction

Logistic regression model	LMA change ≦−6.3% at POY0.5^a^			LMA change ≦−6.4% at POY3.0^b^		
Variable	OR	95% CI	*P* values	OR	95% CI	*P* values
Age ≥65	0.66	0.36–1.18	0.157	1.25	0.72–2.18	0.423
Male	0.75	0.39–1.42	0.372	1.32	0.71–2.44	0.380
Body mass index ≤18.5 kg/m2	0.58	0.23–1.47	0.255	1.65	0.68–3.97	0.267
Smoking history	0.98	0.52–1.86	0.954	1.35	0.73–2.49	0.333
FEV 1% < 70%	0.96	0.53–1.75	0.895	0.69	0.39–1.24	0.212
%VC < 80%	4.97	0.50–49.5	0.171	4.12	0.42–40.9	0.227
Other cancer history	0.54	0.26–1.09	0.085	1.16	0.59–2.31	0.663
Chronic obstructive pulmonary disease	1.98	0.68–5.75	0.209	1.77	0.62–5.08	0.287
Coronary disease	0.31	0.09–1.11	0.071	1.86	0.55–6.24	0.315
Charlson co‐morbidity index ≧1	1.54	0.79–3.02	0.204	0.92	0.49–1.75	0.807
Lobectomy (vs. segmentectomy)	1.12	0.65–1.95	0.685	1.04	0.61–1.78	0.879
Open thoracotomy (vs. cVATS)	4.35	2.57–7.35	<0.001	2.33	1.42–3.82	<0.001
Postoperative complications ≥G2	2.01	1.12–3.61	0.019	1.75	1.00–3.07	0.049
Adjuvant chemotherapy	0.80	0.34–1.87	0.607	0.99	0.44–2.24	0.986

CI, confidence interval; cVATS, complete video‐assisted thoracoscopic surgery; FEV1%, forced expiratory volume in 1 s/forced vital capacity ratio; LMA, latissimus dorsi muscle area; OR, odds ratio; POY, post‐operative year; VC, vital capacity.

^a^ Mean LMA change at POY0.5.

^b^ Mean LMA change at POY3.

**Figure 3:**
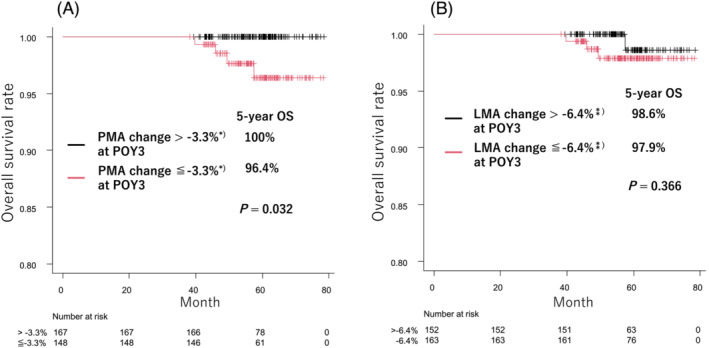
Impact of long‐term postoperative PMA (A) or LMA (B) changes on OS. The 5‐year OS rate of patients with PMA change >−3.3%* group was significantly better than that of patients with PMA change ≤−3.3%* group (*Figure* 3A). There was no difference in the 5‐year OS rate of patients in the LMA change >−6.4%^⁑^ group and LMA change ≤−6.4%^⁑)^ group (*Figure* 3B). *Mean PMA change at POY0.5. ^⁑^Mean LMA change at POY3. LMA, latissimus dorsi muscle area; OS, overall survival; PMA, psoas muscle area; POY, postoperative year.

Four deaths occurred in this study, all of whom underwent lobectomy. The 5‐year OS rate of patients with PMA change >−3.3% group was significantly better than that of patients with PMA change ≤−3.3% group (100.0% vs. 96.4%, *P* = 0.032, *Figure* 3A); however, there was no difference in the 5‐year OS rate of patients in the LMA change >−6.4% group and LMA change ≤−6.4% group (98.6% vs. 97.9%, *P* = 0.366, *Figure* 3B).

**Figure 4:**
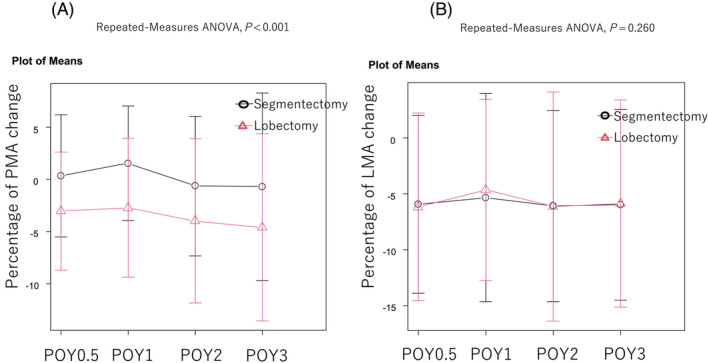
Comparison of PMA (A) and LMA (B) changes between lobectomy and segmentectomy groups after propensity matching. The mean postoperative PMA change was significantly smaller in the segmentectomy group than in the lobectomy group during the postoperative observation period after propensity matching (*Figure* 4A). The mean postoperative LMA change during the postoperative observation period in patients who underwent segmentectomy was not significantly different from that in patients who underwent lobectomy after propensity score matching (*Figure* 4B). ANOVA, analysis of variance; LMA, latissimus dorsi muscle area; PMA, psoas muscle area; POY, postoperative year.

Patient characteristics of lobectomy and segmentectomy groups were comparable after propensity score matching (*Table* 4). Patients who underwent segmentectomy had significantly smaller changes of PMA than those who underwent lobectomy during the postoperative observation period, after propensity score matching (*P* < 0.001, *Figure* 4A). The mean change in the LMA during the postoperative observation period in patients who underwent segmentectomy was not significantly different from that in patients who underwent lobectomy (*P* = 0.428, *Figure* 4B).

**Table 4:** Comparison of patient characteristics between lobectomy and segmentectomy groups after propensity score matching

Total *n* = 150	Lobectomy (*n* = 75)	Segmentectomy (*n* = 75)	*P* values	Stand diff
Age ≥65, No. (%)	57 (76.0)	58 (77.3)	1.000	0.032
Male, No. (%)	33 (44.0)	36 (48.0)	0.743	0.080
Body mass index ≤18.5 kg/m^2^, No. (%)	5 (6.7)	5 (6.7)	1.000	<0.001
Smoking history, No. (%)	44 (58.7)	43 (57.3)	1.000	0.027
FEV1% < 70%, No. (%)	18 (24.0)	20 (26.7)	0.851	0.061
%VC < 80%, No. (%)	0 (0)	0 (0)	NA	<0.001
CT tumour size >2.0 cm, No. (%)	27 (36.0)	28 (37.3)	1.000	0.028
Other cancer history, No. (%)	18 (24.0)	22 (29.3)	0.580	0.121
Charlson co‐morbidity index ≧1, No. (%)	38 (50.7)	40 (53.3)	0.870	0.053
Open thoracotomy, No. (%)	42 (56.0)	47 (62.7)	0.506	0.136
Postoperative complications ≥G2, No. (%)	14 (18.7)	16 (21.3)	0.839	0.067
Adjuvant chemotherapy, No. (%)	0 (0)	0 (0)	NA	<0.001
Adenocarcinoma, No. (%)	67 (89.3)	69 (92.0)	0.780	0.092
Pathological stage I ≥, No. (%)	74 (98.7)	74 (98.7)	1.000	<0.001

CT, computed tomography; FEV1%, forced expiratory volume in 1 s/forced vital capacity ratio; NA, not available; VC, vital capacity.

## Discussion

This is the first study to demonstrate that segmentectomy is less invasive than lobectomy in terms of changes in muscle mass. Postoperative PMA change was significantly smaller in the segmentectomy group than in the lobectomy group. In the lobectomy group, PMA reduction progressed over the long postoperative period, whereas segmentectomy maintained the postoperative psoas muscle mass. Loss of the psoas muscle at POY3 results in poor prognosis in patients who undergo surgery for early‐stage lung cancer. The risk factors for psoas muscle mass loss on POY3 were lobectomy and open surgery, suggesting that segmentectomy via cVATS was associated with a lower likelihood of sarcopenia progression in patients with early‐stage NSCLC after surgery. Latissimus dorsi muscle mass decreased persistently without a significant difference in the PMA change between the lobectomy and segmentectomy after propensity matching. cVATS prevents loss of the latissimus dorsi muscle; however, the prevention of the latissimus dorsi muscle does not seem to affect the prognosis of patients after lung cancer surgery.

Decreased skeletal muscle mass is an important element of sarcopenia. In 2018, the European Working Group on Sarcopenia in Older People defined sarcopenia using three elements: loss of muscle mass, muscle strength and quality of life (QOL).^11^ Sarcopenia increases the risk of cardiac disease, respiratory disease, mobility disorders and cognitive impairment, leading to lower QOL and death.^11^ Recently, the psoas muscle index (PMI), which can be calculated from the PMA easily measured by CT, has been reported to reflect skeletal muscle mass and is known as a surrogate marker of sarcopenia that can predict the prognosis of patients with lung cancer.^14^, ^17^, ^18^ In patients with resectable lung cancer, preoperative sarcopenia, defined by the PMI, is associated with postoperative complications of lung cancer surgery,^14^ recurrence^18^ and postoperative survival.^14^, ^17^, ^19^ Moreover, the PMI is also reported to be associated with prognosis after chemotherapy and programmed cell death protein 1 inhibitor therapy for advanced‐stage lung cancer.^24^, ^25^

Recently, several studies have reported postoperative skeletal muscle loss and survival after surgery. Park et al. reported that a decrease in PMA of 10% or more was a negative prognostic factor for OS in patients with surgically treated oesophageal cancer.^26^ Nagata et al. reported that a decrease in skeletal muscle of ≥9.9% at 6 months postoperatively was an independent prognostic factor for outcome after NSCLC surgery.^27^ Takamori et al. reported that a 10% or more decrease in Th12 skeletal mass index 1 year after surgery from the preoperative skeletal mass index was associated with poor disease‐free survival and OS after surgery.^7^ They also reported that the risk factors for a 10% or more reduction in the skeletal mass index were poor performance status ≥1 and FEV1% < 70%^7^; however, they did not analyse the surgical procedure. The present study showed that segmentectomy avoided persistent postoperative loss of psoas muscle mass, as observed with lobectomy. Moreover, segmentectomy through cVATS may reduce the transition to sarcopenia in the long‐term after surgery.

Recently, the JCOG 0802 trial revealed fewer non‐lung cancer deaths in the segmentectomy group than in the lobectomy group, although lung cancer deaths were similar in both the groups.^4^ Although segmentectomy was considered less invasive than lobectomy in the trial, preservation of FEV1 in the segmentectomy group 1 year after surgery was 3.5% compared with the lobectomy group, which did not reach the predefined threshold for clinical significance of 10%.^4^ Moreover, several studies showed nonsuperiority in the preservation of respiratory function in the long‐term period after segmentectomy compared with lobectomy.^9^, ^10^ Kobayashi et al. reported that the decrease in vital capacity, FEV1, and FEV1% at POY5 from POY1 did not differ between the segmentectomy and lobectomy groups.^9^ Suzuki et al. reported that the respiratory function of patients at ≥13 months after segmentectomy and lobectomy was similar to that of the preoperative period, and no difference was observed between the two groups.^10^ Until now, few studies have physiologically explained the lower invasiveness of segmentectomy. Stamatis et al. reported less frequent deterioration to the baselines of physical and cognitive functioning, dyspnoea and fatigue at 12 months with segmentectomy than with lobectomy, and recovery of dyspnoea was reported to be faster.^28^ The present study suggests that segmentectomy preserves the psoas muscle and, thus, preserves the postoperative QOL of patients with early‐stage NSCLC, which may result in the avoidance of the progression of sarcopenia that may occur after lobectomy.

In this study, cVATS was associated with preservation of the psoas and latissimus dorsi muscle mass in the long postoperative period compared with lobectomy. Similar to our study, Karasaki et al. previously reported that cVATS preserved the reduction of the latissimus dorsi mass more than open thoracotomy at 1 year after surgery: 43% reduction of the latissimus dorsi mass with open thoracotomy and 11% with cVATS on the surgical side.^29^ cVATS has been reported to be a technique that maintains fewer respiratory complications, shorter hospital stay, lower mortality, less pain and better postoperative QOL than open chest surgery without compromising oncological outcomes.^30^, ^31^, ^32^, ^33^ Charloux et al. reported that the postoperative loss of FEV1 was 9% for segmentectomy through an open thoracotomy and 5% for segmentectomy through cVATS.^8^ In the present study, open thoracotomy was associated with reduction of PMA and LMA in the long postoperative period, suggesting that cVATS is a less invasive approach for sarcopenia in patients with NSCLC than open thoracotomy.

The definition of sarcopenia in patients with NSCLC varies from one report to another. The PMA measured at the L3 level using CT is commonly used.^14^, ^17^, ^18^, ^34^, ^35^ Suzuki et al. reported that sarcopenia, defined by the skeletal muscle mass measured at the L3 level, including the psoas, erector spinae, quadratus lumborum, transversus abdominis, external and internal obliques, and rectus abdominis, has been reported to be associated with prognosis.^23^ Sun et al. showed that sarcopenia, defined by the pectoralis muscle index and peak expiratory flow rate, is associated with the postoperative prognosis of NSCLC.^36^ In this study, the OS of patients with PMA change ≤−3.3% at POY3 was significantly worse than those with PMA change >−3.3% at POY3; however, LMA change did not influence OS. The latissimus dorsi muscle, a respiratory supportive muscle, participates in thoracic and brachial motion and is active during deep inspiration and forceful respiratory functions, such as coughing.^37^ Maintaining the function of the latissimus dorsi muscle may be involved in maintaining respiratory function and patients' QOL after thoracic surgery^29^; however, it does not improve prognosis.

This study had several limitations. First, this was a single‐centre retrospective study with a small number of patients. Selection bias may be possible between patients who underwent lobectomy and those who underwent segmentectomy, even with propensity‐matching analysis. Further large‐scale prospective multicentre studies are necessary. Second, this study excluded patients with recurrent NSCLC and those with missing CT data for a certain period within the third postoperative year. Third, this study did not assess muscle strength, patient QOL or prognosis after surgery. Further prospective studies are necessary to examine whether segmentectomy could prevent the progression to sarcopenia better than lobectomy.

This study demonstrated that the psoas muscle mass was better maintained during the postoperative period by segmentectomy than by lobectomy after propensity matching. The decrease in the psoas muscle mass was progressed over the long postoperative period after lobectomy, whereas segmentectomy maintained the postoperative psoas muscle mass. Loss of the psoas muscle after surgery results in a poor prognosis in patients who undergo surgery for early‐stage NSCLC. The risk factors for the loss of psoas muscle mass in the long‐term postoperative period were lobectomy and open surgery, suggesting that segmentectomy via cVATS was associated with a lower likelihood of sarcopenia progression in patients with early‐stage NSCLC after surgery.

## Conflict of interest

None declared.

## Supporting information


**Supporting Information S1.** Codes for mixed ANOVA using EZR on R Commander.
**Supporting information S2.** Codes for propensity score matching using EZR on R Commander.

**Data S1.** Supplementary Information.
