# Supplementary material for: Long‐term changes in psoas muscle mass after lobectomy and segmentectomy for early‐stage lung cancer
Source: J Cachexia Sarcopenia Muscle. 2023 Sep 23;14(6):2540–9. doi: 10.1002/jcsm.13328 (PMC10751413; doi:10.1002/jcsm.13328)
Supplement: Supplementary file 1 — Supporting Information S1. Codes for mixed ANOVA using EZR on R Commander. Supporting information S2. Codes for propensity score matching using EZR on R Commander. [file JCSM-14-2540-s002.docx]

**Supplemental information 1: Codes for mixed ANOVA using EZR on R Commander**

The following codes were used to conduct a mixed ANOVA generated by EZR on the R commander:

TempDF <- Dataset

TempDF$Factor1.B解析5..術式.0.区切以下..1.葉切以上 <- factor(TempDF$B解析5..術式.0.区切以下..1.葉切以上)

contrasts(TempDF$Factor1.B解析5..術式.0.区切以下..1.葉切以上) <- "contr.Sum"

#Convert to long format to draw graph

n <- length(TempDF[,1])

TempDF$TempID <- c(1:n)

TempDF2 <- data.frame(TempID=TempDF$TempID, AAA腸POY0.5=TempDF$AAA腸POY0.5,

AAA腸POY1=TempDF$AAA腸POY1, AAA腸POY2=TempDF$AAA腸POY2, AAA腸POY3=TempDF$AAA腸POY3,

B解析5..術式.0.区切以下..1.葉切以上=TempDF$B解析5..術式.0.区切以下..1.葉切以上)

TempDF2 <- na.omit(TempDF2)

TempDF3 <- reshape(TempDF2, idvar="TempID", varying=list(c("AAA腸POY0.5",

"AAA腸POY1", "AAA腸POY2", "AAA腸POY3")), v.names="data", direction="long")

RepeatNumber <- c("AAA腸POY0.5", "AAA腸POY1", "AAA腸POY2", "AAA腸POY3")

nvar <- length(TempDF3$time)

for (i in 1:nvar){TempDF3$time2[i] <- RepeatNumber[TempDF3$time[i]]}

windows(width=7, height=7); par(lwd=1, las=1, family="sans", cex=1, mgp=c(3.0,1,

0))

StatMedplotMeans(TempDF3$data, factor(TempDF3$time2),

factor(TempDF3$B解析5..術式.0.区切以下..1.葉切以上), error.bars="sd", xlab="", ylab="",

legend.lab="B解析5..術式.0.区切以下..1.葉切以上", , lty=1, lwd=1)

AnovaModel.18 <- lm(cbind(AAA腸POY0.5, AAA腸POY1, AAA腸POY2, AAA腸POY3) ~

Factor1.B解析5..術式.0.区切以下..1.葉切以上, data=TempDF, na.action=na.omit)

time <- factor(c("AAA腸POY0.5", "AAA腸POY1", "AAA腸POY2", "AAA腸POY3"))

time <- data.frame(Time = time)

res <- NULL

res <- Anova(AnovaModel.18, idata=time, idesign=~Time, type="III")

summary(res, multivariate=FALSE)

**Supplemental information 2: Codes for propensity score matching using EZR on R Commander**

Codes for propensity score matching were provided by the “Program of IPTW and PS Matching,” purchased from the Web (<http://rcommander.cart.fc2.com/>) of Research Mind Corporation (Osaka, Japan).
