# Supplementary material for: Long‐term changes in psoas muscle mass after lobectomy and segmentectomy for early‐stage lung cancer
Source: J Cachexia Sarcopenia Muscle. 2023 Sep 23;14(6):2540–9. doi: 10.1002/jcsm.13328 (PMC10751413; doi:10.1002/jcsm.13328)
Supplement: Supplementary file 2 — Data S1. Supplementary Information. [file JCSM-14-2540-s001.docx]

**Supplementary Tables** Details of lobectomy and segmentectomy

| Procedure | Side | Location | n |
| --- | --- | --- | --- |
| Lobectomy | Right | Upper | 80 |
|  |  | Middle | 25 |
|  |  | Lower | 38 |
|  | Left | Upper | 43 |
|  |  | Lower | 36 |
| Segmentectomy | Right | S1 | 3 |
|  |  | S2 | 6 |
|  |  | S3 | 5 |
|  |  | S2-S6 | 1 |
|  |  | S5 | 1 |
|  |  | S6 | 12 |
|  |  | S8 | 2 |
|  |  | S8+9 | 1 |
|  |  | S10 | 1 |
|  |  | S9+10 | 1 |
|  | Left | Upper segment | 27 |
|  |  | S1+2 | 8 |
|  |  | S1+2+6 | 1 |
|  |  | S3 | 3 |
|  |  | Lingular segment | 11 |
|  |  | S6 | 4 |
|  |  | S8 | 1 |
|  |  | S10 | 4 |
|  |  | Basal segment | 1 |
